# Broad vs. narrow traits: a scoping review of measuring personality traits in teacher selection using the situational judgment test

**DOI:** 10.3389/fpsyg.2023.1217321

**Published:** 2023-09-29

**Authors:** Azad Iqram Nadmilail, Mohd Effendi Ewan Mohd Matore, Siti Mistima Maat, Lynn Sheridan

**Affiliations:** ^1^Faculty of Education, The National University of Malaysia (UKM), Bangi, Selangor, Malaysia; ^2^Research Centre of Education, Leadership and Policy, Faculty of Education, The National University of Malaysia (UKM), Bangi, Selangor, Malaysia; ^3^University Research Groups (KPU), Educational Evaluation, The National University of Malaysia (UKM), Bangi, Selangor, Malaysia; ^4^STEM Enculturation Research Center, Faculty of Education, The National University of Malaysia (UKM), Bangi, Selangor, Malaysia; ^5^Faculty of the Arts, Social Sciences and Humanities, School of Education, University of Wollongong, Wollongong, NSW, Australia

**Keywords:** broad traits, narrow traits, teacher selection, situational judgment test, personality traits, scoping review

## Abstract

Situational Judgment Tests (SJTs) have gained popularity and are commonly used as a measurement technique in a variety of professions, particularly those that include hiring, promoting, and professional development. In various educational sectors around the world, SJTs are being utilized as a measure by which to choose individuals who possess the requisite non-academic attributes for the profession. The objective of this review is to identify and analyze the traits that are measured in teacher selection using SJTs, in terms of both broad and narrow traits. This review uses a scoping review approach comprising five stages which are: identifying the research question, identifying relevant studies, selecting the studies, charting the data and reporting the results. Seven empirical research studies on teacher selection using SJTs were identified in which broad and narrow traits are used differently in selection. In the studies, a broad trait—conscientiousness—and seven narrow traits—organization and planning; empathy and communication; adaptability and resilience; mindset; emotional regulation; professional ethics; and enthusiasm and motivation—were recognized. Analysis revealed, in line with other prior studies, that the traits discovered tended to be used as the foundation for teacher selection criteria. The use of broad and narrow traits as the basis for teacher selection criteria has implications for the selection of the “best” teacher candidates because both broad and narrow traits generally do not accurately measure the precise characteristics needed. Future studies should focus on measuring more precise characteristics without overlap between the targeted characteristics, in light of the conclusions from this review.

## 1. Introduction

The growing acceptance of Situational Judgment Tests (SJTs) as a psychological and employability measuring tool in many businesses throughout the world has accelerated their adoption for selection and promotional purposes. SJTs are very popular and widely used in many fields such as education, medicine, nursing, and the military (Nadmilail and Mohd Matore, 2021). SJTs are also utilized in personnel selection and measuring candidates’ performance for promotion (Whetzel et al., 2020). Personnel selection is viewed as a priority when selecting new candidates for a profession and is often the first measure used to determine suitability for entry (Nadmilail et al., 2022a). For example, the field of medicine in the United Kingdom has established that selection is the first procedure to be carried out in medical education and training (Patterson et al., 2016). In this instance SJTs are an important first tool in the selection of medical personnel with the necessary traits needed for success in the medical role. Similarly, in the United States military, with a high level of competition among newly appointed officers for promotion, SJTs are used to promote military leaders based on candidates’ leadership judgments (Lievens et al., 2008; Whetzel et al., 2020). SJTs are an important first tool to measure not only the selection of new personnel but also for promotional purposes. A number of SJT-related studies have been undertaken in Western countries, they include in the United States (Feranchak and Deiger, 2017; Erickson and Herbst, 2018), the United Kingdom (Elliott et al., 2011; Klassen et al., 2014, 2016, 2020; Kim and Klassen, 2018), and Germany (Gold and Holodynski, 2015, 2017; Aldrup et al., 2020; Gold et al., 2021).

Most studies utilizing SJTs have measured the psychology of test candidates based on hypothetical situations to describe the interpersonal, intrapersonal, and intellectual constructs (National Research Council, 2015). As SJTs gain popularity as a predictor of personnel performance, various organizations around the world have begun to utilize SJTs as an individual psychological and employability measurement (Weekley et al., 2013). The main challenge in utilizing SJTs as a measurement tool is in accurately predicting a person’s work performance and suitability based on the outcome of a targeted scenario response during the selection process (Nadmilail et al., 2022a). However, prediction based on SJTs has been notably helpful in educational psychology research, as well as explanations of the success of university students (Breen and Lindsay, 2002; Matošková and Kovářík, 2017). The development of SJTs can be supported by having a full understanding of workplace circumstances relevant to workers’ accomplishments. In certain instances, the workplace can provide extra contextual information regarding the right personality qualities and measurements to use when selecting people (Golubovich et al., 2020). Consequently, SJTs are increasingly being used as a measurement tool worldwide in various professions, for various purposes such as: personnel selection (Klassen et al., 2014, 2016, 2020; Al Hashmi and Klassen, 2019; Wang et al., 2023), recruitment (Gold and Holodynski, 2015, 2017; Gold et al., 2021), and professional development (Aldrup et al., 2020; Pavlidou and Alevriadou, 2022).

For the purpose of this review, which focuses on teacher education and non-academic traits, the review will look specifically at utilizing SJTs in selecting distinct broad and narrow traits in teacher selection identified in the literature over the last 10 years. Numerous studies on teacher personnel selection have been undertaken over the years. For instance, Klassen and Kim (2019) reviewed the literature and discovered 32 studies that were published between 2000 and 2017. The review explained selection methods other than the use of SJT. Anglim and O’Connor (2018) explained broad traits as those relating to a general personality predisposition (i.e., conscientiousness and extraversion) leading to abstract behaviors and experiences. Meanwhile, Paunonen et al. (2003) described narrow traits, as traits that offer a larger variety of criteria and result in specific behavioral acts (e.g., organization, diligence, perfectionism and prudence). Therefore, this review will look at how broad and narrow traits are categorized in research. Measuring non-academic traits based solely on the IQ test achievement can be limiting when assessing candidates for a profession or promotion. As such, SJTs offer an additional tool to accurately assess relevant non-academic traits in clinical and professional practice, particularly interpersonal traits like integrity, empathy and resilience, which are seen as critical for success in the workplace (Patterson et al., 2012; Sheridan et al., 2022a). Hence, understanding how, personality dispositions (broad traits) and traits asresulting in behavior acts (narrow traits) are utilized in teacher education to assess candidates’ suitability can help stakeholders and researchers to determine what extra contextual information and individual behavior is relevant when making teacher selections.

## 2. Literature review

### 2.1. Situational Judgment Tests

According to Lievens and Coetsier (2002), SJTs are utilized as a measurement method to evaluate the respondents’ view or interpretation of a work-related environment that describes a real work scenario. SJTs also aim to measure competence and interpersonal traits related to work (Lievens et al., 2008). This methodology is designed to measure the non-academic traits of the targeted characters (Patterson and Driver, 2018). Importantly, SJTs are a valuable instrument used to determine psychological measures of candidates that can contribute a critical lens and knowledge to enable employers to make decisions and evaluate results based on the responses given by test candidates (Nadmilail and Mohd Matore, 2021). Moreover, SJTs are one of the best predictive measurement methods as they offer a range of measurement strategies with varying test structures (Ployhart, 2013).

Situational Judgment Tests are also known as a simulation method (O’Connell et al., 2007) that requires respondents to make judgments in situations that highlight problems in certain tasks (Al Hashmi and Klassen, 2019). The simulation test contains a set of actual-state tasks requiring them to respond as if they were doing the tasks. This reaction is interpreted as an indication of the expectation of future behavior, which is particularly important for professions such as medicine, education, and the military. Generally, the fidelity of the test shows a simulation that varies depending on how the test is performed (Nadmilail and Mohd Matore, 2021). When the test is used as an accurate expectation of the actual working situation, this indicates that the simulation test has a high level of fidelity. SJTs are useful in describing actual workplace situations (e.g., misbehavior in a classroom) and are designed to create a contextualized individual assessment of a specific situation within the workplace environment (Ryan and Ployhart, 2014). By linking the SJT scenario to work-related situations, a collection of critical situations and responses provides an important link to the context. In this way, the SJTs are used to target specific traits or competencies based on task descriptions, with the selected response a predictor of possible actions that can be taken based on the description of the targeted task.

The development of SJTs instruments are based on several theories, various viewpoints and opinions related to SJTs used. For example, Motowidlo et al. (2006) focused on Behavioral Consistency Theory related to SJTs. This theory describes past behavior as the best predictor of future behavior. The main principle of this theory highlights current behavioral samples to allow for the prediction of future behavior (Motowidlo et al., 2006). SJTs have also been evidenced to serve as a good predictor of work performance because the instrument measures the procedural understanding of effective behavior in a particular situation (Lievens and Patterson, 2011). Thus, the predictions shown by the test candidates can provide a clearer picture to the selection panel as a key indicator for the decision-making process regarding selection, promotion, and professional development needs.

Motowidlo et al. (2013) elaborated further using Implicit Trait Policy (ITP) that focuses on implicit motivational actions. The ITP entails implicit beliefs about the consequences of various actions pertaining to the effectiveness of such actions (Motowidlo et al., 2013). These actions are measured in SJTs as the main function along with the behavioral characteristics of the choice of responses and the individuals’ consideration of the effectiveness of their behavior. However, responses can depend on specific areas such as job level, job knowledge and job description (Motowidlo and Beier, 2010; Patterson et al., 2016), and in certain situations, measure internal traits and/or “behaviors” (Golubovich et al., 2020; Hejri et al., 2023). Furthermore, tendencies or traits that have been patterned can contribute to general ideas about required behaviors and characteristics, with individuals having different beliefs about the effectiveness of certain behaviors related to inherent tendencies or personality traits.

In the field of teacher education research, numerous empirical studies have been conducted using SJTs for various purposes include personnel selection (Klassen et al., 2014, 2016, 2020; Al Hashmi and Klassen, 2019), personnel promotion (Gold and Holodynski, 2015, 2017; Gold et al., 2021) and professional development (Aldrup et al., 2020; Pavlidou and Alevriadou, 2022). The utilization of SJTs, in education policies or practice is often based on the requirements of stakeholders. For example, the aim of the education policy at the international level is to produce quality teachers who can deliver high-quality teaching and improve student outcomes (Beauchamp et al., 2013). As such, the focus on teacher selection is often about both attracting as many candidates as possible and selecting quality teacher candidates (Feuer et al., 2013; Schleicher, 2014; Nadmilail et al., 2023). It is important to note, however, that personnel selection procedures often require cultural suitability and the need to be responsive to evolving and changing organizational needs which reflect organizational strategy and the available jobs (Nadmilail et al., 2022b).

Several recent reviews involving the selection of teaching personnel have been conducted in various countries. For instance, Klassen and Kim (2019), conducted a review that measured academic and non-academic traits as well as the effectiveness of teachers using other methods, locating a total of 32 studies overall. Nadmilail and Mohd Matore (2021) conducted a review regarding country-of-origin and time trends utilizing SJTs; they identified 20 specific studies in eight countries, including: England (6), Germany (4), Greece (2), Australia (2), the United States (2), Ireland (1), Taiwan (1), and Oman (1). Nadmilail et al. (2022a) also reviewed seven studies focusing on intrapersonal and interpersonal traits measured, utilizing SJTs in teacher education. Nadmilail et al. (2022a) review identified eight interpersonal traits—organization, planning empathy, communication, teaching, relationships with colleagues, counseling, and contingency. Meanwhile seven intrapersonal traits were also identified—conscientiousness, mindset, emotion regulation, adaptability, enthusiasm and motivation, resilience, and professional ethics. However, none of those reviews looked specifically at broad and narrow traits, the focus of this review.

In this review, we provide a compelling rationale for examining and comparing broad and narrow features that have been overlooked in previous publications. The existing literature has significantly contributed to our understanding of the topic at hand, but there is a critical gap in the exploration of these particular issues. We can contribute to new knowledge and progress in this field by conducting a comprehensive study that analyzes both general and specific features. This comparative approach will allow us to identify previously unexplored relationships, patterns, and differences. Furthermore, by addressing this research gap, we hope to achieve a more comprehensive understanding of the topic. The inclusion of both broad and narrow features will allow us to uncover nuanced details and complexities that may have been overlooked in previous research, leading to a more complete and accurate representation of the topic. Furthermore, this comparative analysis will allow us to close the existing knowledge gap and create a more robust and comprehensive research corpus. This will not only enrich academic discourse, but also have practical implications for practitioners, policymakers, and other stakeholders who rely on accurate and comprehensive information. In summary, the significant gap in the literature regarding the study of broad and narrow characteristics warrants this review. By addressing this gap, we can provide new insights, advance the field, and provide a more comprehensive understanding of the topic, thereby enriching academic discourse and supporting practical applications.

### 2.2. Defining broad and narrow traits

The current review aims to provide a nuanced understanding of teacher selection for non-academic traits by identifying and analyzing the traits as either broad or narrow. In general, non-academic traits are often associated with personality (Niessen and Meijer, 2016). Personality can be differentiated into two types: broad traits and narrow traits (Paunonen et al., 2003; Aksin, 2023). The main aspects being measured in teacher selection include intellectual attributes and personality. Intellectual attributes represent an academic (cognitive) trait, while personality is viewed as a non-academic trait. There are various ways to label non-academic traits (e.g., intrapersonal, interpersonal, broad, narrow). Anglim and O’Connor (2018) defined broad traits as general personality tendencies toward abstract behaviors and experiences. Dwivedi et al. (2009) elaborated broad traits as global constructs representing relatively enduring characteristics of individuals consistent over time and across situations. Broad traits are those that represent the sum of numerous lower-level personality traits or facets (Paunonen et al., 2003; McKinney et al., 2023). Meanwhile narrow traits are able to account for greater criteria variance and particular behavioral acts (Paunonen et al., 2003). Dwivedi et al. (2009) explained that narrow traits are conceptually specific constructs, often components of broad traits, that are consistent over time and across situations. Meanwhile Anglim and O’Connor (2018) defined narrow traits as a specific personality tendency toward concrete behaviors and experiences. Narrow traits also have value for research and practice beyond the broad traits (Credé et al., 2016).

Various definitions have been elaborated by researchers about broad and narrow traits. In short, broad traits are persistent features of humans that persist across time and contexts, as well as incorporate a number of lower-level personality traits. In contrast, narrow traits are precise personality dispositions toward tangible behaviors, theoretically specific constructs that are continuous, and accountable for more behavioral criteria in terms of diversity and specificity. Differences between broad traits and narrow traits as identified in the literature are depicted in Table 1.

**TABLE 1:** Differences between broad traits and narrow traits.

Model	Broad traits	Narrow traits
Neo-PI-R model Costa and McCrae (1992) –This study provides a model to specify the range of traits that a comprehensive personality instrument should measure	Neuroticism	Anxiety, hostility, depression, self- consciousness, impulsiveness, vulnerability to stress
	Extraversion	Warmth, gregariousness, assertiveness, activity, excitement seeking, positive emotion
	Openness to experience	Fantasy, aesthetics, feelings, actions, ideas, values
	Agreeableness	Trust, straightforwardness, altruism, compliance, modesty, tender mindedness
	Conscientiousness	Competence, order, dutifulness, achievement
Big five aspects DeYoung et al. (2007)—This study indicates the existence of two distinct aspects within each of the big five inventory	Neuroticism	Withdrawal, volatility
	Extraversion	Enthusiasm, assertiveness
	Openness to experience	Openness/creativity, intellect
	Agreeableness	Politeness, compassion
	Conscientiousness	Orderliness, industriousness
HEXACO-PI-R model Ashton et al. (2014)—This study discusses the misunderstood aspects of HEXACO that have been adapted and adopted by several studies especially on (H) honesty-humility, (A) agreeableness and (E) emotionality factors	Honesty-humility	Sincerity, fairness, geed avoidance, modesty
	Emotionality	Fearfulness, anxiety, dependence, sentimentality
	Extraversion	Social Self-Esteem, Social boldness, sociability, liveliness
	Agreeableness	Forgiveness, gentleness, flexibility, patience
	Conscientiousness	Organization, diligence, perfectionism, prudence
	Openness to experience	Aesthetic Appreciation, Inquisitiveness, Creativity, Unconventionality
Big five inventory 2 (BFI-2) (Soto and John, 2017)—This study extended, discussed and revised the big five inventory (BFI)	Extraversion	Sociability, assertiveness, energy level
	Agreeableness	Compassion, respectfulness, trust
	Conscientiousness	Order, self-discipline, dutifulness
	Negative emotionality	Anxiety, depression, emotional volatility
	Open-mindedness	Intellectual curiosity, aesthetic sensitivity, creative imagination

Four main models are outlined which relate to non-academic traits. In each of the models the traits are divided into those considered broad and narrow traits. The Neo-Pi-R Model pioneered by Costa and McCrae (1992) show five domains with 30 facets used for the basis of research on personality structure and development. The second model is the Big Five personality model. DeYoung et al. (2007) model is comprised of five domains with 10 facets which extend the investigation of level of organization within the Big Five by addressing some of the limitations of past studies. Additionally, Ashton et al. (2014) pioneered the HEXACO-PI-R Model, which comprises six domains with 25 facets which discuss the misunderstood aspects of HEXACO that had been adapted and adopted by several studies especially on (H) honesty-humility, (A) agreeableness and (E) emotionality factors. Meanwhile Soto and John (2017) Big Five Inventory 2 (BFI-2) model consists of five domains with 15 facets which extended, discussed and revised the Big Five Inventory (BFI).

Although lexical studies have focused primarily on the number of traits and broad traits, the analysis of large item group factors has also proven that traits can be represented in terms of nested hierarchies. The hierarchical model of personality usually consists of one or more broad or general characteristics at the highest level, with other characteristics that are more specific or narrow and include more traits at a lower level. In such models, a broad set of traits consists of several aspects or facets that help define a broad nature and provide a perspective that is more related to the personality.

## 3. Methodology

The current study adopted a scoping review approach based on Arksey and O’Malley (2005) as its methodology, and applied a review protocol that determines the topic to be studied. A scoping review is useful in gaining a comprehensive overview of a broad issue and in charting existing research to better prepare for future research (Munn et al., 2018). A scoping review is useful for analyzing developing evidence when it is unclear whether other, more specific questions may be presented and addressed profitably by a more precise systematic review (Armstrong et al., 2011). As such, this methodology was chosen as it helped to synthesize all the relevant academic literature, with a specific focus on detail. Arksey and O’Malley (2005) methodology included the following five steps: (1) identify the research question; (2) identify relevant studies; (3) study selection; (4) chart the data; and (5) collate, summarize, and report results. It was also necessary to report on the types of evidence that addressed and informed field practice (e.g., Lundmark et al., 2021).

Step 1—identifying the research aim and establishing the research questions. Our research aims to examine the literature to identify and describe the characteristics of non-academic traits—broad and narrow traits measured in teacher selection using SJTs. The following research questions pose as a means of directing the search for relevant literature in teacher education selection:

1. What non-academic traits are measured in teacher selection using SJTs?
2. Does trait selection take broad and narrow traits into account as key factors?
3. How are broad and narrow traits determined?

**FIGURE 1:**
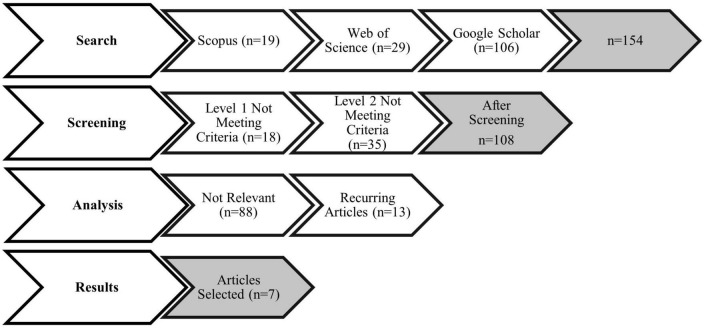
Adaptation of the article selection process from Karabulut-ilgu et al. (2018).

Following the identification of the research questions is step 2: the identification of relevant studies. In the select of relevant studies, four phases were utilized based on the method proposed by previous studies (Karabulut-ilgu et al., 2018; Zainal and Mohd Matore, 2019; Nadmilail and Mohd Matore, 2021; Nadmilail et al., 2022a) as shown in Figure 1. The four phases of this review include the search phase, the screening phase, the analysis phase, and the results phase. In this review, all articles were identified with the keywords “Situational Judgment Tests” and “Teacher Selection.” In general, SJTs were used in this study to provide a comprehensive overview of the characteristics used in teacher selection based on a scoping analysis of relevant publications.

This phase involves an article search strategy using a search database that focuses on three major databases in the scientific world, namely Web of Science (WoS), SCOPUS, and Google Scholar. Web of Science was chosen because it has become one of the world’s leading scientific citation, discovery and analytical information search platforms used as an academic library research tool, as well as a rich data set for large-scope information across diverse academic fields (Li et al., 2017). Meanwhile, SCOPUS was considered because it is progressively used in academic papers (only slightly less than its competitors, Web of Science) and it challenges the external Web of Science section (Zhu and Liu, 2020). In addition, Google Scholar can offer important resources for publicly accessible archives, covering a wide range of disciplines and languages that are unmatched in the efficient and effective preparation of online scientific documents (Gusenbauer, 2019). In this phase, the keywords “Situational Judgment Tests” and “Teacher Selection” were used. The appropriate keywords were selected based on the goals that the search was intended to achieve. The researchers also used the phrase search function and the Boolean operator OR or/and AND, to combine the keywords in the initial search phase. In this research, three basic techniques were used for manual searching, namely hand picking, backward tracking, and forward tracking (Mohamed Shaffril et al., 2020). A search strategy was then added to obtain the most recent articles, and finally the relevant articles were selected by limiting the year of publication to January 2012 to December 2021 (i.e., within 10 years).

After all the articles were identified, the next step, step 3, was to select the studies. To obtain accurate and appropriate articles, several screening steps were performed on the initial articles, as described in Table 2. The two acceptance criteria included: (1) journal articles only and (2) from a period between January 2012 and December 2021, while the two rejection criteria included: (1) systematic literature studies and (2) articles in languages other than English. The next strategy involved removing recurring articles by reading the titles and abstracts. The final analysis was done through a close reading of the remaining articles to remove articles that were not relevant to the requirements of the study. After completing step 3, the selection of literature, the fourth step consisted of the graphical presentation of the selected articles. Summaries were created for each article based on the author, year, and title of the selected study. Based on the search, filtering, and analysis processes, a total of seven articles were selected. The seven selected articles are shown in Table 3. The fifth and final phase of this review framework involved compiling, summarizing, and reporting on the results. All data were stored and processed in Microsoft Excel. A synthesis of the literature was created by summarizing the individual points and presenting them in text, tables, and figures.

**TABLE 2:** Screening criteria.

General criteria	Acceptance criteria	Rejection criteria
Year of publication	January 2012–December 2021	Other than January 2012–December 2021
Type of publication	Empirical review articles	Other than empirical review articles
Language	English	Other than English

**TABLE 3:** Summary of selected articles.

References	Country	Study focus
Bardach et al., 2021b	England	This study focuses on adverse effects of gender, ethnicity, and socio-economic status (SES) on SJT scores, by exploring both main effects and interactions, and considering both overall SJT performance and separate SJT domain scores.
Bardach et al., 2021a	England	This study examines whether video-based SJT formats provide benefits over “traditional” text-based SJTs.
Klassen et al., 2020	England	This study focuses on development and testing of online SJTs designed to screen applicants for invitation to an ITE interview day.
Chao et al., 2020	Taiwan	This study develops a series of SJTs to make up for the inadequacies of using academic performance exclusively as the admission criterion for student teachers.
Al Hashmi and Klassen, 2019	Oman	This study develops SJTs for selecting ITEP applicants in Oman, explores its psychometric properties and explores applicants’ reactions to the test.
Klassen et al., 2016	England	This study reports on the development of proof-of- concept SJTs to assist in the selection of candidates for primary teacher education (ITE) programs.
Klassen et al., 2014	England	This study designs a pilot situational judgment test (SJTs) for selection into primary and secondary teacher training programs in the UK by considering applicants’ perceptions.

## 4. Findings

The focus of this review is to identify the characteristics of non-academic traits, involving broad and narrow traits, that are measured in empirical studies on teacher selection. The findings revealed seven research articles that have a significant relationship because of a basis in the topic SJTs and schoolteachers have been identified based on the keywords “Situational Judgment Tests” and “Teacher Selection.” This review provides a valuable contribution by expanding our understanding of the distinction between broad and narrow traits in situational judgment testing in teacher education.

### 4.1. Non-academic traits measured in teacher selection

This review analyzed the characteristics of broad and narrow traits that are measured in teacher selection literature utilizing SJTs. There are key distinctions between the ways in which broad and narrow traits are applied during the teacher selection process in the literature. Firstly, the selection of broad or narrow traits relies upon different organizational or individual needs and traits and their importance for the job role. While narrow traits are specific and concrete, broad traits are identified as more general and abstract. Broad and narrow traits in teacher selection utilizing SJTs are shown in Table 4.

**TABLE 4:** Non-academic traits measured.

References	Broad trait	Narrow traits
Bardach et al., 2021b	Conscientiousness	Mindset, emotion regulation
Bardach et al., 2021a	Conscientiousness	Organization and planning, empathy and communication, adaptability and resilience, mindset, emotion regulation
Klassen et al., 2020	Conscientiousness	Organization and planning, empathy and communication, adaptability and resilience, mindset growth, emotion regulation
Chao et al., 2020	Not discussed	Classroom management, teaching, relationships with colleagues, parent-teacher communication, counseling, contingency
Al Hashmi and Klassen, 2019	Not discussed	Communication, organization and planning, resilience and adaptability, professional ethics, enthusiasm and motivation
Klassen et al., 2016	Not discussed	Organization and planning, empathy and communication, adaptability and resilience
Klassen et al., 2014	Not discussed	Organization and planning, empathy and communication, adaptability and resilience

Three studies (Klassen et al., 2020; Bardach et al., 2021a,b) focused on conscientiousness as a broad trait with a specific focus on and interest in teacher effectiveness and classroom performance. The studies suggest that conscientiousness is important as a comprehensive trait because it is closely related to job performance and effectiveness in the classroom. Conscientious teachers are likely to be more organized, responsible, and effective with their classes and students, ultimately leading to better educational outcomes for their students. Conscientious teachers plan their lessons more carefully, are punctual and reliable, and take responsibility for their students’ academic success. They are also more likely to discipline themselves, be persistent, and manage their time effectively—all important skills for teachers who must juggle multiple tasks and responsibilities. Kim et al. (2018) explained that conscientiousness is important as a goal attribute because it has been shown to be one of the Big Five personality domains most strongly related to teacher effectiveness.

Meanwhile all of these studies identified mindset and emotional regulation as important narrow traits to be measured. Mindset and emotional regulation are important characteristics to measure when selecting prospective teachers because they are closely related to effective teaching practices and student outcomes. Mindset refers to a person’s belief system and attitude toward learning and intelligence. Teachers who have a growth mindset, meaning they believe intelligence and skills can be developed through effort and practice, are more likely to create a positive and supportive learning environment for their students. They are also more likely to set high expectations for their students and encourage them to take risks and learn from their mistakes. Emotion regulation refers to a person’s ability to manage their emotions and respond appropriately to different situations. Teachers who have good emotional regulation skills are better able to cope with the stress and demands of the teaching profession, which can be challenging and emotionally draining at times. They are also better able to address the emotional needs of their students and create a supportive and positive learning environment. Teachers who have problems with emotional regulation are more prone to burnout or emotional exhaustion, which can negatively impact their ability to teach effectively and provide a positive learning experience for their students. According to Seaton (2018) study, mindset or mindset growth was chosen because of the increasing recognition that teachers’ beliefs have an impact on how students perceive their learning. Meanwhile, in Taxer and Gross (2018) study, emotional regulation was chosen because teacher emotions, and emotional regulation are tied to a variety of essential teaching-related outcomes.

Klassen et al. (2014, 2016, 2020), Al Hashmi and Klassen (2019), and Bardach et al. (2021a) identified organization and planning, empathy and communication and adaptability and resilience as narrow traits needed for selecting prospective teachers because these are essential skills that contribute to effective classroom practice and positive student outcomes. Effective teachers must be well organized and able to plan effectively to manage their time, resources, and lesson plans. Teachers must also be able to communicate effectively and empathetically with their students to create a supportive and inclusive learning environment. There are also essential skills that enable teachers to respond effectively to the challenges and changes that occur in the classroom and in the education system in general. Teachers must be able to adapt to changing circumstances and show resilience in the face of adversity. Organization and planning were highlighted because both traits could measure the ability to manage competing goals, demonstrate strong time management and personal organizational abilities, and foster positive learning relationships with students, as noted in Klassen et al. (2016) study. In Klassen et al. (2018) study, empathy and communication focused on actively listening and communicating with students and colleagues, aggressively seeking professional and student feedback, as well as the ability to adjust speech and discourse. Meanwhile, in Klassen et al. (2014) study, adaptability and resilience highlighted the ability to adapt lessons and sequence, self-awareness and confidence to ask for support or make decisions alone, and accept criticism and acknowledge difficulties.

Chao et al. (2020) study identified no broad trait but did identify five narrow traits which included: classroom management, teaching, relationships with colleagues, parent-teacher communication, counseling and contingency. In this study, all of the traits had been chosen because those traits were seen as important aspects for the professional teacher to demonstrate. Finally in Al Hashmi and Klassen (2019) study no broad trait was identified; however, two narrow traits were identified as important: professional ethics, and enthusiasm and motivation. According to Al Hashmi (2018), professional ethics is important as teachers must be prepared and able to take on the moral responsibility as a role model for young people in their classroom. To do this, they need to show they need to be trustworthy, honest and respectful. In this study, enthusiasm and motivation are strongly associated with teacher effectiveness, with teacher enthusiasm positively predicting students’ interest.

### 4.2. Broad and narrow teacher traits as key factors

Based on the review, researchers combined both characteristics with specific purposes based on the objectives of the study, as the objectives are usually local in nature and focused on the needs of specific countries. In most cases, the decision on which characteristics to measure was based on the consensus opinion of the appointed experts. Al Hashmi and Klassen (2019) focused on the development of an SJT for the selection of Initial Teacher Education Programs (ITEPs) applicants in Oman, as well as the study of psychometric properties and applicant responses to the test. Chao et al. (2020) conducted research in Taiwan and developed a set of SJTs to compensate for the shortcomings of using only academic achievement as an admission criterion for student teachers. In addition, the selected characteristics reflected the socioeconomic characteristics of the study area. In the study by Bardach et al. (2021b), there was also clear evidence of the relationship between gender, ethnicity, and socioeconomic status (SES) and SJT outcomes. Both main effects and interactions were examined, and both overall SJT performance and individual SJT domain outcomes were considered. In this study, the researchers did not characterize the characteristics as broad and narrow, as this was not deemed necessary to explain the outcomes that meet local needs.

Each study also utilized different methods and techniques to determine the selected traits. Bardach et al. (2021a,b) studies included conscientiousness based on empirical quantitative evidence from Klassen et al. (2020), which has shown that teachers who scored higher in conscientiousness were likely to perform better in the classroom (Kim et al., 2019). This is similarly to the narrow traits identified based on a previous study (Klassen et al., 2020). Subsequently, Klassen et al. (2020) selected conscientiousness because it is one of the domains in the Big Five Personality which is the dominant personality model and most related to teacher effectiveness (Kim and Maccann, 2018). In Klassen et al. (2020) study, narrow traits that are considered foundational were found by combining inductive and deductive methods during the content development process. This was done to match important events with the area of interest and then to assign categories to the content using inductive methods. This approach involved an integrated “construct-informed” inductive and deductive approach in which features were developed from a series of expert interviews and based on existing theories. Klassen et al. (2014) developed those traits through discussions with expert teachers which involved administering Initial Teacher Education (ITE) selection activities, usually Initial Teacher Training mentors working closely with trainee teachers using the critical incident technique.

## 5. Discussion

### 5.1. Broad traits

The aim of the review was to provide a nuanced understanding of the non-academic characteristics of faculties in the selection of teachers, which are described in the literature as either broad or narrow. To this end, the study sought to determine which non-academic characteristics were measured in the selection of teachers using SJTS, whether broad and narrow characteristics were considered in the selection process, and finally, how broad and narrow characteristics are determined in the literature. Only three papers identified broad traits. Table 5 illustrates the findings from Klassen et al. (2020) and Bardach et al. (2021a,b), indicating only one broad trait identified—conscientiousness. Klassen et al. (2020) stated that conscientiousness was chosen as a target trait because it is the strongest personality predictor of job performance, is most strongly related to teacher effectiveness among the Big Five personality domains, and is one of the strongest predictors of overall teacher evaluations. The main justification given by Bardach et al. (2021a,b) for choosing conscientiousness as the measurable trait is also this assertion.

**TABLE 5:** Summary of findings on broad trait.

Broad trait references	Conscientiousness
Bardach et al., 2021b	x
Bardach et al., 2021a	x
Klassen et al., 2020	x
Total	**3**

Stoll et al. (2020) defined conscientiousness as a tendency to always be careful, punctual, rule-following, and diligent. This trait encompasses personality components such as self-sufficiency, regularity, responsibility, achievement, self-discipline, and prudence (Costa and McCrae, 1992). In addition, people in this group are always ambitious and strive to excel in everything they do. The trait of conscientiousness was selected to measure the self-efficacy of individuals considered suitable for selection as teachers. It is also consistent with McCrae and Costa (2006), DeYoung et al. (2007), and Ashton et al. (2014) definition of conscientiousness as being efficient, reasonable, rational, neat, punctual, and organized, with great ambition and a desire for perfection. Conscientiousness has the potential to be developed based on explanations from different researchers with different others narrow traits. Although the developed traits are generally characterized, the selection of traits should be based on suitability, especially with regard to job tasks. Therefore, conscientiousness can be described as self-sufficiency, regularity, a sense of duty, willingness to perform, self-discipline, and prudence. Conscientiousness in teachers represents a state in which thoughts, feelings, and behaviors matter, and this trait has the potential to distinguish one person from others (Turiano, 2020). In general, teachers with high levels of conscientiousness have the ability to orient, control, and exhibit responsible behavior (Djigić et al., 2014; Aksin, 2023). They also tend to plan wisely and are able to adhere to established rules or norms (Abd Rahman, 2010). It could be assumed that a group of teachers who are conscientious tend to have a high level of competence and rationality in their decision making (Erjavec et al., 2019). This is critical for teachers and instruction, in order to provide engaging learning activities for students and to create high-quality instruction.

Teachers who possess this personality trait have a high value of conscientiousness and are always careful when making decisions, especially when it comes to teaching, learning, and student achievement. In fact, conscientiousness shows the ability of teachers to act in accordance with their minds. Those who are chosen as teachers must have a high level of awareness and be constantly aware of their surroundings. They must also always be careful when making a decision to avoid jeopardizing students’ futures by making the wrong choices and to ensure that teaching and learning can be done well and effectively. This situation is closely related to wise planning, which is also a description of conscientiousness. Therefore, it is very important to ensure that those selected have a high level of conscientiousness. As conscientiousness has become more important in the selection of teachers worldwide, this characteristic is necessary to measure teacher effectiveness. It is critical to include conscientiousness in teacher selection because it is related to teacher effectiveness, and conscientiousness is a malleable personality trait (Robinson, 2009). Clearly, ITE needs individuals with high levels of conscientiousness to be trained, especially in planning high-quality teaching and learning. They must also adhere to established rules or standards and be aware of professional expectations. Professional expectations require teachers to act as role models for students because the school community, such as parents and colleagues, expect professionalism from teachers.

In summary, conscientiousness was selected to measure the self-efficacy of individuals considered suitable for selection as teachers. Teachers who possess this personality trait have a high sense of values and show diligence in all decisions concerning, in particular, teaching, learning, and student achievement. In fact, conscientiousness indicates the ability of teachers to act in accordance with their consciousness (Wimmer et al., 2019). It is important to include conscientiousness as a comprehensive trait in teacher selection.

### 5.2. Narrow traits

Table 6 shows a summary of narrow characteristics in teacher selection using SJTs in the literature. A total of six studies focused on organization and planning, empathy and communication, and adaptability and resilience (Klassen et al., 2014, 2016, 2020; Al Hashmi and Klassen, 2019; Chao et al., 2020; Bardach et al., 2021a,b). Klassen et al. (2014) explained that organization and planning aim to measure the candidate’s ability to manage competing priorities and effectively demonstrate time management and personal organization skills to enhance positive learning interactions with students. In Chao et al. (2020), organization and planning were not clearly stated; however, the characteristics identified include classroom management and instruction, which can be categorized under organization and planning. Organization and planning are also specific, narrow characteristics of the trait of conscientiousness (Ashton et al., 2014; Soto and John, 2017).

**TABLE 6:** Summary of findings on narrow traits.

References narrow trait	Organization and planning	Empathy and communication	Adaptability and resilience	Mindset	Emotion regulation	Professional ethics	Enthusiasm and motivation
Bardach et al., 2021b	x	x	x	x	x		
Bardach et al., 2021a	x	x	x	x	x		
Klassen et al., 2020	x	x	x	x	x		
Chao et al., 2020	x	x	x				
Al Hashmi and Klassen, 2019	x	x	x			x	x
Klassen et al., 2016	x	x	x				
Klassen et al., 2014	x	x	x				
Total	**6**	**6**	**6**	**3**	**3**	**1**	**1**

Klassen et al. (2016) describe that organization and planning, which measure candidate ability, have similarities to organization in the classroom, with the dimensions of behavior management and teaching-learning formats. In teaching and learning, organization and planning are considered key requirements for instructional success (Sheridan et al., 2022b). Organization and planning are especially important in ensuring student participation and engagement in classroom activities (Pavlidou and Alevriadou, 2022). In addition, teachers who provide clear instructions and demonstrate the ability to organize classroom activities are essential. Moreover, these skills are very important in demonstrating knowledge of different methods of assessing student learning, such as formative and summative tests, diagnostic methods, and formal methods. Consequently, these qualities are always an important factor in teacher selection. Organization and planning are also important, as they are key prerequisites for employability (Zakaria et al., 2014). Both characteristics are also used as important benchmarks to ensure that the selected personnel are of good quality (Nadmilail et al., 2022a). To preserve the quality of the selected personnel, ITE could provide the selected teachers with theoretical and practical training on classroom management and the role of teachers in managing regular and inclusive classes. They should also be exposed to models of discipline management, dealing with problematic behavior in the classroom, and creating an effective classroom management plan.

Empathy and communication have also been identified as important teacher characteristics. Meyers et al. (2019) define teacher empathy as how well teachers understand students’ personal and social conditions and how carefully they manage students’ emotions. Dhillon and Kaur (2021) describe teacher communication as a form of psychological control over students that has a significant impact on their achievement and academic success. Klassen et al. (2014) explain teacher empathy and communication as teachers’ ability to actively listen and engage in open dialog with both students and colleagues. These elements are important to promote meaningful and guided learning in the classroom. Indeed, a good teacher-student relationship can help students feel comfortable and engage more deeply with the learning content. Klassen et al. (2014) see empathy and communication as related traits that are evident in the ability to proactively seek advice and respond to professional feedback and student needs. Chao et al. (2020) developed these traits by incorporating communication with parents and counseling sessions, which helped teachers accept opinions from students and colleagues to improve teaching practices. Teachers should have the ability to communicate and collaborate effectively with different parties in a global, economic, environmental, and community context. This element is also closely related to the ability to adjust communication style accordingly (Klassen et al., 2014). Therefore, it is very important to test, measure and develop these narrow characteristics. ITE providers could teach effective communication skills to pre-service teachers so they can use a range of verbal and non-verbal communication strategies, especially to promote student engagement. Dor (2018) suggests that formal training should be provided to address such issues in the classroom. In addition, they need to be taught a high level of empathy, especially in preparation for dealing with real-life situations in school involving students with disabilities and diversity in the community. Certainly, effective communication skills and a high level of empathy can undoubtedly be achieved by prospective teachers.

Adaptability and resilience are specific features of the trait “agreeableness,” as described by Ashton et al. (2014) and Costa and McCrae (1992). Collie and Martin (2016) initially define adaptability as an individual’s ability to adapt to environmental opportunities and challenges over the course of a lifetime. Adaptability is also defined as the ability to make changes according to a particular aptitude based on a particular situation (Klassen et al., 2014). Seligman and Csikszentmihalyi (2014) describe resilience as a dynamic process that enables constructive adaptation in the face of adversity. Ryan and Ployhart (2014) defined adaptability as a person’s ability, skills, propensity, willingness, and motivation to change or adapt to various tasks and social and environmental characteristics. Sheridan et al. (2022a) noted that adaptability and resilience are combined because adaptability is considered a protective factor for resilience that is related to a person’s personal profile (e.g., skills, personality). Klassen et al. (2014) specified adaptability and resilience in six characteristics: (1) the ability to remain resilient under pressure; (2) the demonstration of adaptability; (3) the ability to change teaching methods as needed; (4) the wise application of appropriate levels of competence; (5) the willingness to accept challenges and critical feedback; and (6) the appropriate use of a variety of effective strategies. These six aspects are explained in detail by Durksen and Klassen (2018) who refer to the ability to change instruction and the instructional sequence appropriately when needed, the confidence to make decisions independently, the willingness to accept challenges to one’s knowledge, and the use of appropriate coping strategies. In addition, Chao et al. (2020) did not use adaptability and resilience as traits in the context of agreeableness, where unexpected contingencies were used in the study. However, unexpected contingencies can pose challenges to adaptability and resilience. Unexpected contingencies such as natural disasters, pandemics, or cyberattacks can disrupt normal operations and require organizations and individuals to adapt quickly to new circumstances. In the context of the teaching profession, adaptability and resilience are necessary for staff selection because teachers must balance their duties and personal lives in a professional manner (Nadmilail et al., 2022a). Teachers face various challenges on a daily basis, whether at school or in their daily lives. As a result, teachers must always maintain a high level of adaptability and resilience to effectively manage any critical circumstances. ITE must also ensure that all programs and courses offered include elements to strengthen the adaptability and resilience of prospective teachers. Even if they pass the test during the selection process, reinforcement must be provided to ensure they are truly prepared for real-world situations. Sheridan et al. (2022a) state that building adaptability and resilience requires time and opportunities for self-reflection, as well as supportive formal and informal relationships. This better prepares them for future issues they may face as teachers.

Three studies addressed mindset, or growth mindset, and emotion regulation as key characteristics for teacher selection (e.g., Klassen et al., 2020; Bardach et al., 2021a,b). Mindset refers to the process of developing different meanings, goals, motivations, and behaviors (Schröder et al., 2021). Meanwhile, Seaton (2018) explained that mindset is an individual’s view of intelligence that can be developed and expanded. Mindset was selected as one of the target traits because when teachers believe that learning and students’ abilities are flexible, which this can affect teachers’ teaching as well as students’ achievement and confidence (Nadmilail et al., 2022a). Growth mindset was chosen as a metric because it is increasingly recognized that teacher attitudes impact how students perceive their learning (Klassen et al., 2020). This argument is supported by Seaton (2018) who states that teachers’ personal beliefs and practices are critical in encouraging students to explore their own mindsets and develop thinking practices that promote learning. Therefore, teachers’ attitudes toward themselves and their students play a critical role in setting their expectations, teaching practices, and how students view their own perspectives. A positive attitude ensures that the teaching and learning process runs smoothly by effectively managing stress, setting successful learning goals, and being surrounded by positive people at all times. Since the growth mindset has been used to recognize that teachers’ attitudes influence how students perceive their learning in staff selection, ITE must consider the aspect of reinforcement in training to avoid the occurrence of a fixed mindset in prospective teachers. The characteristics of the fixed mindset are being a quitter, self-centered, fearful, deceptive, and attention-seeking, while the characteristics of the growth mindset are being persistent, determined, diligent, and opportunity-oriented (Monique et al., 2020). Through ITE learning, the growth mindset is seen as malleable by supporting prospective teachers in a clinical instructional model and familiarizing them with the culture of the school. Mindset can be specifically described under the characteristics of openness to experience (Costa and McCrae, 1992; DeYoung et al., 2007; Ashton et al., 2014) and open-mindedness (Soto and John, 2017), both of which are categorized as broad characteristics.

Emotional regulation can be characterized by the traits of neuroticism and extraversion (Costa and McCrae, 1992). Costa and McCrae (1992) explain that the trait neuroticism consists of personality components, namely anxiety, worry, preoccupation, depression, anger, lack of self-control, and vulnerability. Meanwhile, extraversion consists of being friendly, sociable, assertive, fun-seeking, cheerful, and active. Stoll et al. (2020) also explained that neuroticism tends to involve negative emotions such as anger, anxiety, and sadness, and sensitivity in dealing with others. This is in comparison to extraversion, which is a dimension of personality variation that includes individual differences in sociable behavior, assertiveness, and emotions that are always positive, influential, and motivated (Lukaszewski, 2020). Aldrup et al. (2020) describe emotional regulation as the process by which an individual influences his or her emotions and expression of emotions. Koschmieder and Neubauer (2021) define emotional regulation as the ability to change emotions through self-control strategies. In short, emotional regulation can be expressed in positive or negative ways. In the context of the teaching profession, the most common emotional regulation strategies used to regulate teachers’ emotions in the classroom are problem solving, cognitive reappraisal, activity and social support, avoidance, suppression, and rumination (Aldrup et al., 2020). These strategies are thought to be particularly effective for maintaining affective well-being because teachers must deal with emotional situations in their day-to-day work. This job with high emotional demands can be overwhelming and lead to emotional exhaustion (Kim et al., 2019). Therefore, mindfulness training can be implemented in the context of ITE to promote beneficial emotional regulation strategies and decrease depressed mood among prospective teachers (Wimmer et al., 2019). Such training would prepare future teachers for the increased demands in terms of emotional management skills needed to effectively teach and manage their own emotions.

Professional ethics was identified only in the study by Al Hashmi and Klassen (2019), which is seen as a combination of the traits agreeableness (Ashton et al., 2014) and conscientiousness (Soto and John, 2017). In this study, the term “professional ethics” was used as a translation of the Arabic “Akhlaqiyat Al Mihnah,” which is widely used in the education system in Oman and appears in the first statement of the job description for teachers. Peterson and Arthur (2021) defined “ethics” as a moral principle that deals with the advantages or disadvantages of a habit or temperament. Meanwhile, “professionalism” is defined as a privately held belief about one’s behavior as a member of a profession (Burmeister, 2017). Hence, professional ethics refers to the moral principle and conviction of an individual or a group of employees in performing a job. Accordingly, professional ethics were selected to ensure that the selected personnel are noble individuals who adhere to the discipline of the profession (Nadmilail et al., 2022a). In the context of the teaching profession, Nadmilail et al. (2022a) describe professional ethics as a moral quality that is directly related to any type of teaching practice, including concern for students, conscientiousness, determination, and willingness to collaborate with colleagues. Professional ethics or moral qualities are directly related to teaching practice, such as concern for students, enthusiasm for the subject, conscientiousness, determination, willingness to collaborate with colleagues, and a variety of other qualities (Peterson and Arthur, 2021). Therefore, ITE should design and provide appropriate training in the form of practical courses in which practical professional ethics can be well applied so that the aspect of teacher preparedness is no longer a major problem among prospective teachers.

The last group of narrow characteristics included enthusiasm and motivation, which were found to be closely intertwined in the study by Al Hashmi and Klassen (2019). Enthusiasm and motivation were used in this study because they are strongly associated with teacher effectiveness. Enthusiasm can be defined as the pleasure and joy one feels while performing an activity (Lazarides et al., 2018). Meanwhile motivation, on the other hand, according to Heckhausen and Heckhausen (2018), refers to the activation of life orientation toward a positively valued goal. Simply put, enthusiasm and motivation refer to the positive qualities a person needs when doing something to achieve a positive work outcome. Both enthusiasm and motivation are important to ensure that teachers are always passionate about teaching and more supportive of their students (Nadmilail et al., 2022a). A positive attitude can have an impact on student motivation in the classroom. Therefore, enthusiasm and motivation are the qualities that employees must exhibit to gain credibility with the hiring organization (Bougie and Sekaran, 2020), as are the characteristics of extraversion (DeYoung et al., 2007). In relation to the teaching profession, teachers who are enthusiastic in the classroom can increase their students’ motivation by providing championship-oriented activities so that teachers can transfer their genuine enthusiasm to their students (Lazarides et al., 2018). Hence, ITE must select prospective teachers who are motivated to deliver effective instruction that enhances their students’ learning.

## 6. Implications

There are several important implications for the selection of teachers for the teaching profession. The first implication is that there is no concrete definition for the comprehensive characteristic of conscientiousness. Although several conceptualizations of teacher effectiveness have been proposed over the years (e.g., Anwar et al., 2021; Munna and Kalam, 2021), there are different definitions for this term. A clear definition for teacher-awareness selection is important if trait selection is to be based on local needs and guided by local strategies. In all four studies, teacher effectiveness was described in terms of different characteristics based on local needs, and the nature and importance of these characteristics varied by setting (Klassen et al., 2018). Therefore, there is room for improvement in understanding and defining this characteristic and in terms of how it is interpreted globally and locally in education.

The second implication is that trait selection does not establish broad and narrow traits as key factors in practice, while broad traits are a combination of several narrow traits, based on previous findings in other fields (Costa and McCrae, 1992; DeYoung et al., 2007; Ashton et al., 2014; Soto and John, 2017). Organization and planning are narrow traits compared to conscientiousness which is identified as a broad trait in Ashton et al. (2014) and Soto and John (2017). Klassen et al. (2020) and Bardach et al. (2021a,b) and used all three traits in their study. However, the purpose of the characteristic is selected based on the goal and standard of the selection. Conscientiousness was selected because it relates to teacher effectiveness. Organization and planning were selected to measure the applicant’s ability to manage priorities and time. Therefore, the study of teacher selection based on characteristics must be systematic to ensure that there is no overlap or repetition in the characteristics used. However, future studies should be more careful in using appropriate analytical methods to gain clarity about the specific trait or traits measured by the SJT.

The third implication is the development of knowledge about what conscientiousness looks like in practice. Three recent studies (e.g., Klassen et al., 2020; Bardach et al., 2021a,b) have begun to pay attention to this characteristic. Conscientiousness has broad and varied connotations in the literature, explaining several narrow traits (e.g., Costa and McCrae, 1992; DeYoung et al., 2007; Ashton et al., 2014; Soto and John, 2017). However, in the context of teacher selection, this characteristic has been developed under the rubric of teacher efficacy (e.g., Klassen et al., 2020). Although conscientiousness is defined differently in the teaching profession, this trait appears to be stable and malleable (Robinson, 2009) and has the potential to be developed in greater detail. Therefore, future studies could focus on a more detailed definition of conscientiousness by listing characteristics associated with the teaching profession. This may simultaneously innovate and contribute to the field of teaching and future research.

## 7. Conclusion

This review is intended for ITE researchers, stakeholders, and anyone with authority to set standards for research assessment. The review can also assist the ITE sector in redefining the level of its research and alerting assessment authorities to the importance of sector-specific criteria. Thus, the review has brought to light several characteristics that are commonly used in faculty selection as broad and narrow. These characteristics could serve as key factors for selecting the best teachers who are effective and stay in the profession. The broad trait of conscientiousness is useful in predicting teachers’ job performance and effectiveness. Narrow traits, on the other hand, point to more specific and detailed requirements that explain particular traits. Organization and planning are useful in giving precise instructions and demonstrating the ability to organize classroom activities. Empathy and communication skills are useful for actively listening, talking with students and colleagues, seeking direction, and adapting communication styles and discussions. Adaptability and resilience are useful in demonstrating the ability to adjust instruction and sequencing, make independent judgments, and accept gaps in knowledge. Mentality could demonstrate that teacher attitudes influence student learning. Emotional regulation includes teacher problem solving, cognitive processing, activity, social support, avoidance, suppression, and rumination. Professional ethics captures moral qualities related to teaching practice, including concern for students, conscientiousness, determination, and teamwork. Enthusiasm and motivation affirm passion for teaching and provide a support system for students. Originally, researchers chose to use the traits only for narrow characteristics because they were more specific and targeted. However, in line with current developments and the diversity of analysis techniques, broad traits were selected and used so that the traits used for selection are broader and more general to ensure that the selected personnel are at least performing as required. To further enhance the competency of a characteristic, professional development will also be provided to ensure that prospective teachers are better prepared to meet future challenges in the field.

## Author contributions

AN and LS: methodology, formal analysis, investigation, writing—original draft preparation, and project administration. LS: writing—review and editing, visualization and supervision. MM and SM: funding acquisition. All authors contributed to conceptualization, validation, resources, and data curation, read and agreed to the published version of the manuscript.
